# Supplementary material for: The First Optimization Process from Cultivation to Flavonoid-Rich Extract from Moringa oleifera Lam. Leaves in Brazil
Source: Foods. 2022 May 17;11(10):1452. doi: 10.3390/foods11101452 (PMC9140588; doi:10.3390/foods11101452)
Supplement: Supplementary file 1 [file foods-11-01452-s001.zip › foods-1685887-supplementary.pdf]

APPENDIX A: The First Optimization Process from Cultivation to Flavonoid-Rich Extract from *Moringa oleifera* Lam. Leaves in Brazil

Larissa Marina Pereira Silva [1,2,3], Maria Raquel Cavalcanti Inácio [1], Gualter Guenter Costa da Silva [4], Jucier Magson de Souza e Silva [4], Jefferson Romáryo Duarte da Luz [5,6], Maria das Graças Almeida [5], Edgar Perin Moraes [7], Debora Esposito [2], Leandro De Santis Ferreira [3] and Silvana Maria Zucolotto [1,*]

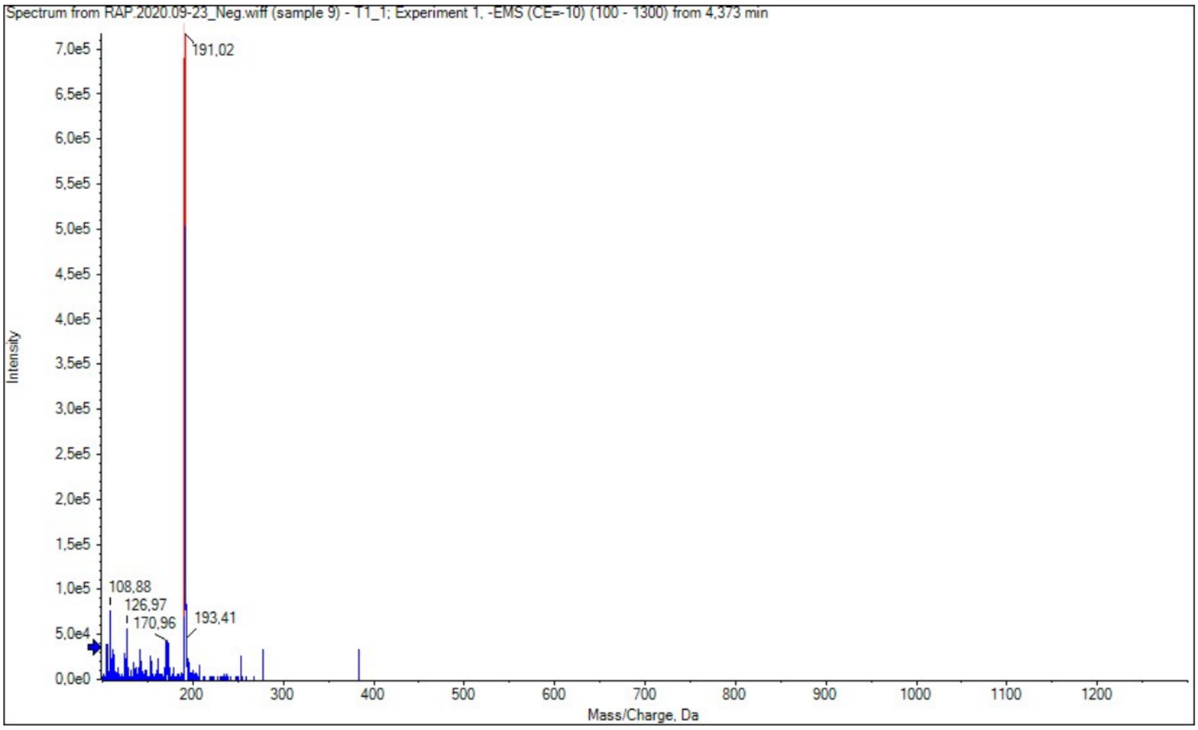

Figure S1. The mass spectrum obtained from flavonoid-rich *M. oleifera* leaf extract (MOL-Flav) in the negative ion mode of peak Nº **1** by HPLC–QTRAP-MS analysis related to the compound quinic acid.

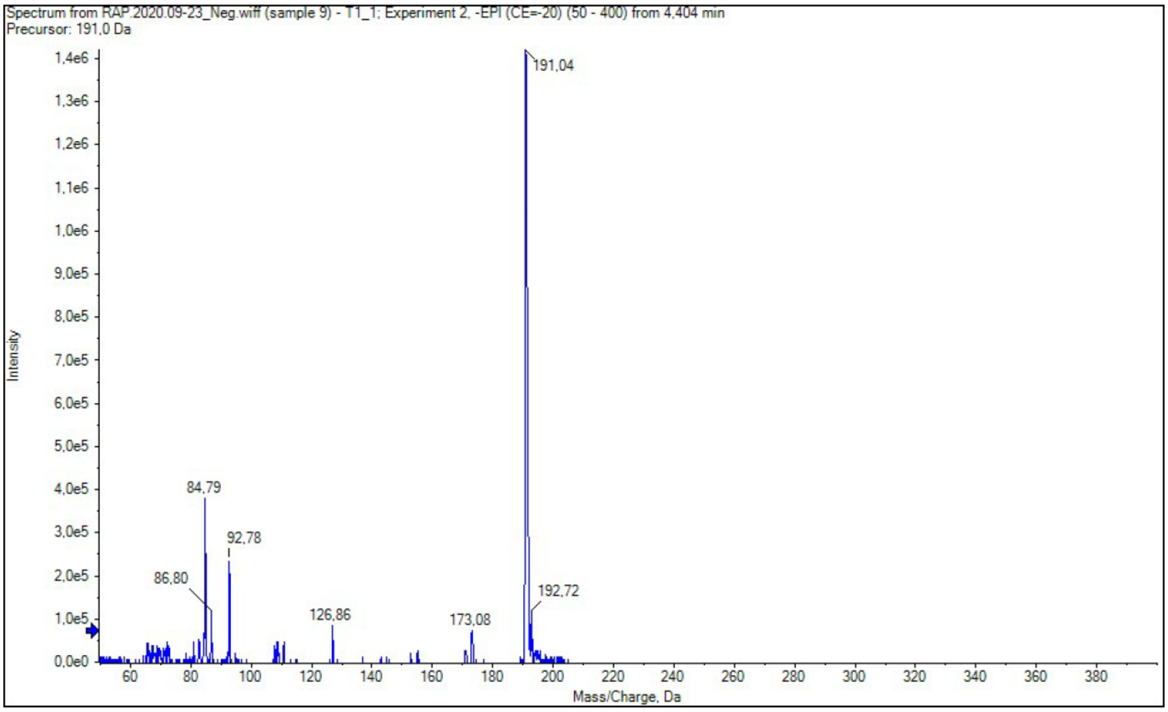

Figure S2. The fragmented mass spectrum obtained from flavonoid-rich *M. oleifera* leaf extract (MOL-Flav) in the negative ion mode of peak Nº **1** by HPLC–QTRAP-MS/MS analysis related to the compound quinic acid.

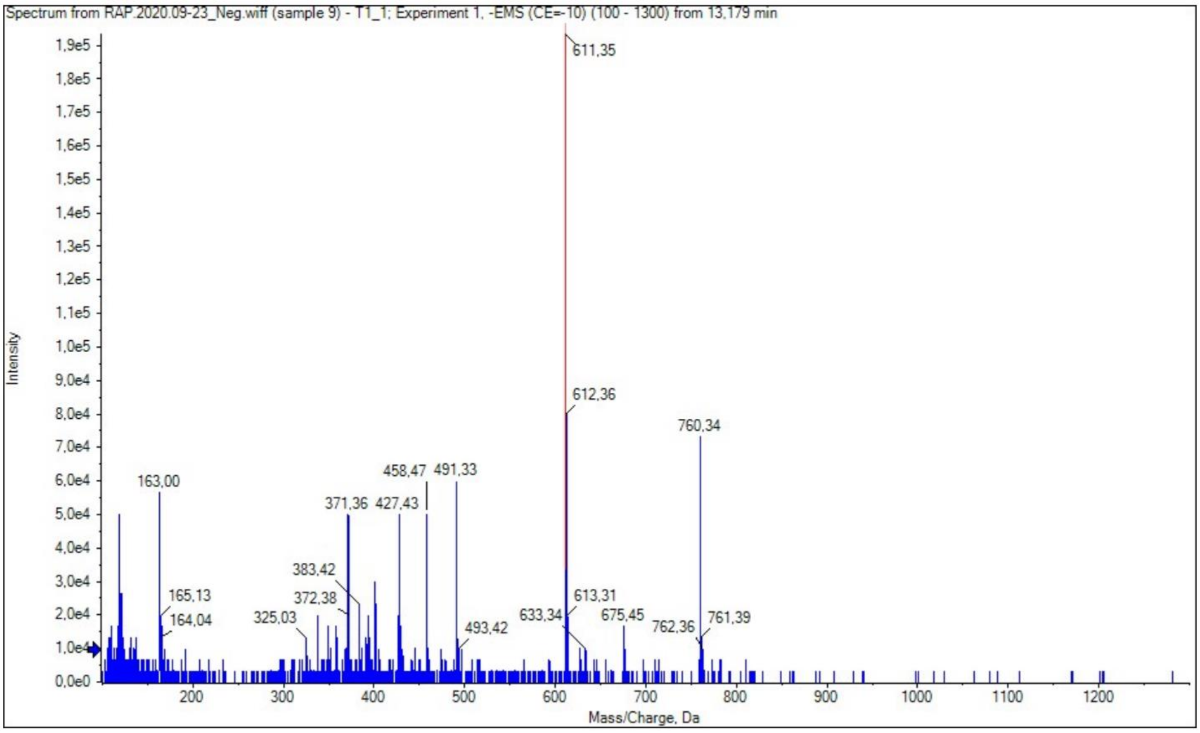

Figure S3. The mass spectrum obtained from flavonoid-rich *M. oleifera* leaf extract (MOL-Flav) in the negative ion mode of peak Nº **2** by HPLC–QTRAP-MS analysis.

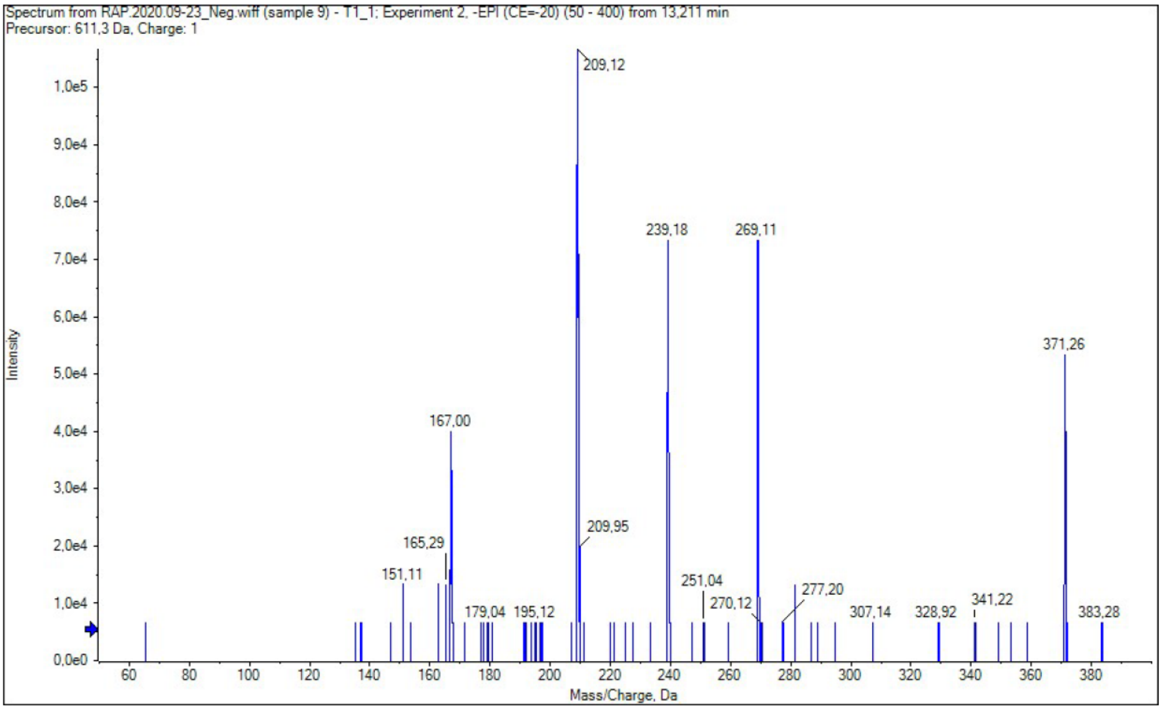

Figure S4. The fragmented mass spectrum obtained from flavonoid-rich *M. oleifera* leaf extract (MOL-Flav) in the negative ion mode of peak Nº **2** by HPLC–QTRAP-MS/MS analysis.

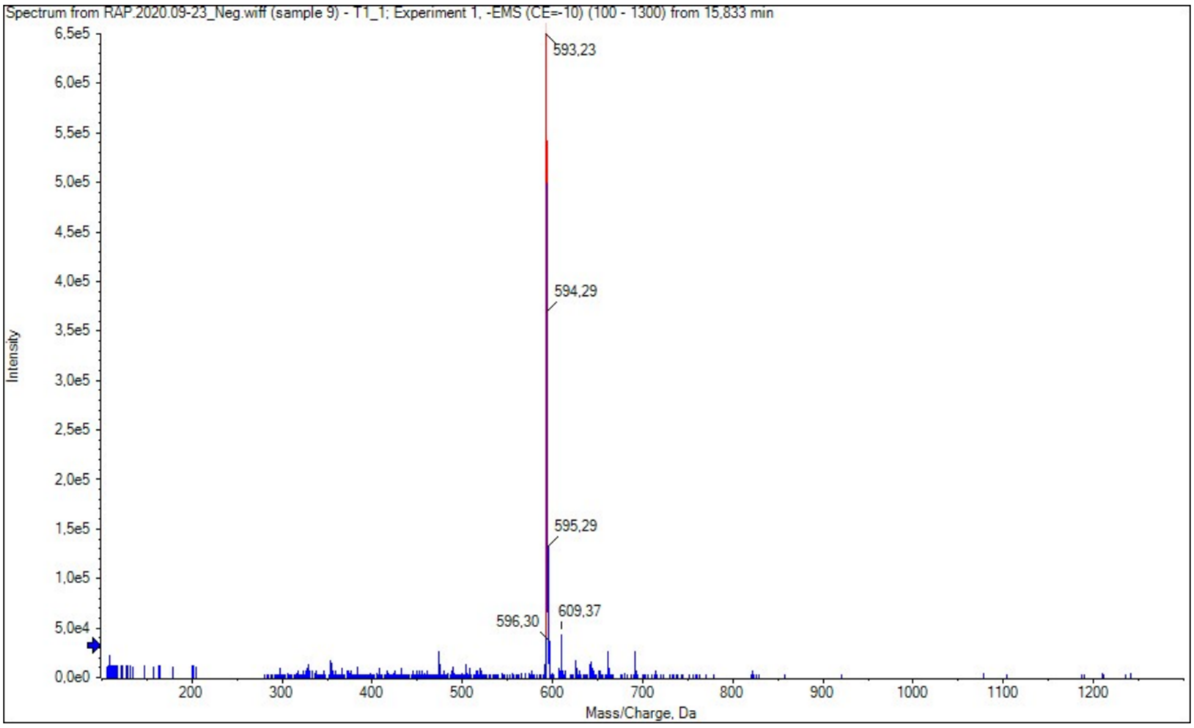

Figure S5. The mass spectrum obtained from flavonoid-rich *M. oleifera* leaf extract (MOL-Flav) in the negative ion mode of peak Nº **3** by HPLC–QTRAP-MS analysis related to the compound Apigenin-6,8 -*C*-dihexose (vicenin-2).

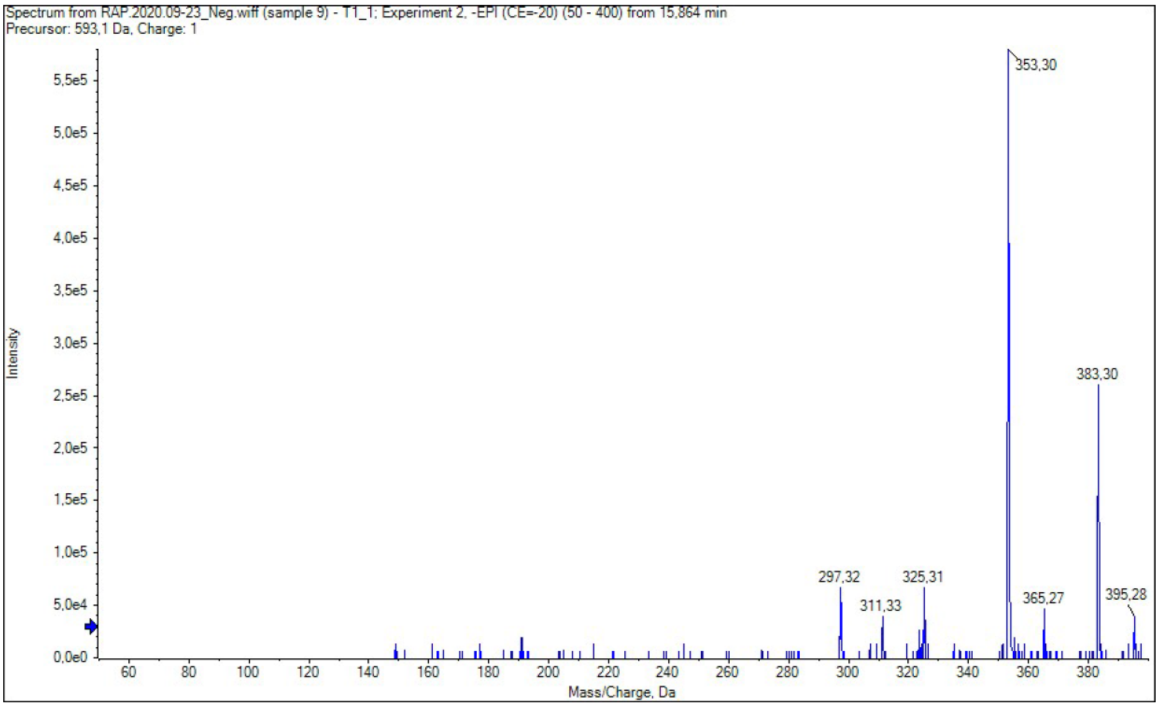

Figure S6. The fragmented mass spectrum obtained from flavonoid-rich *M. oleifera* leaf extract (MOL-Flav) in the negative ion mode of peak Nº **3** by HPLC–QTRAP-MS/MS analysis related to the compound apigenin-6,8 -*C*-dihexose (vicenin-2).

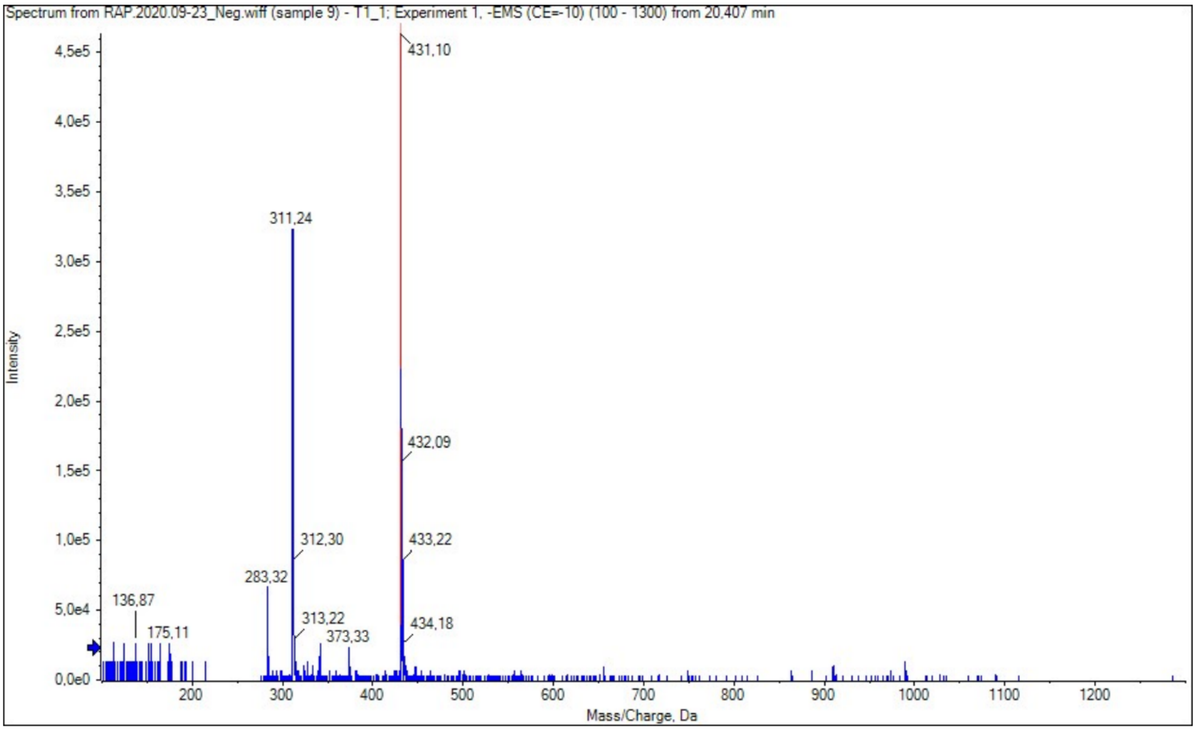

Figure S7. The mass spectrum obtained from flavonoid-rich *M. oleifera* leaf extract (MOL-Flav) in the negative ion mode of peak Nº **4** by HPLC–QTRAP-MS analysis related to the compound apigenin hexose isomer I (vitexin).

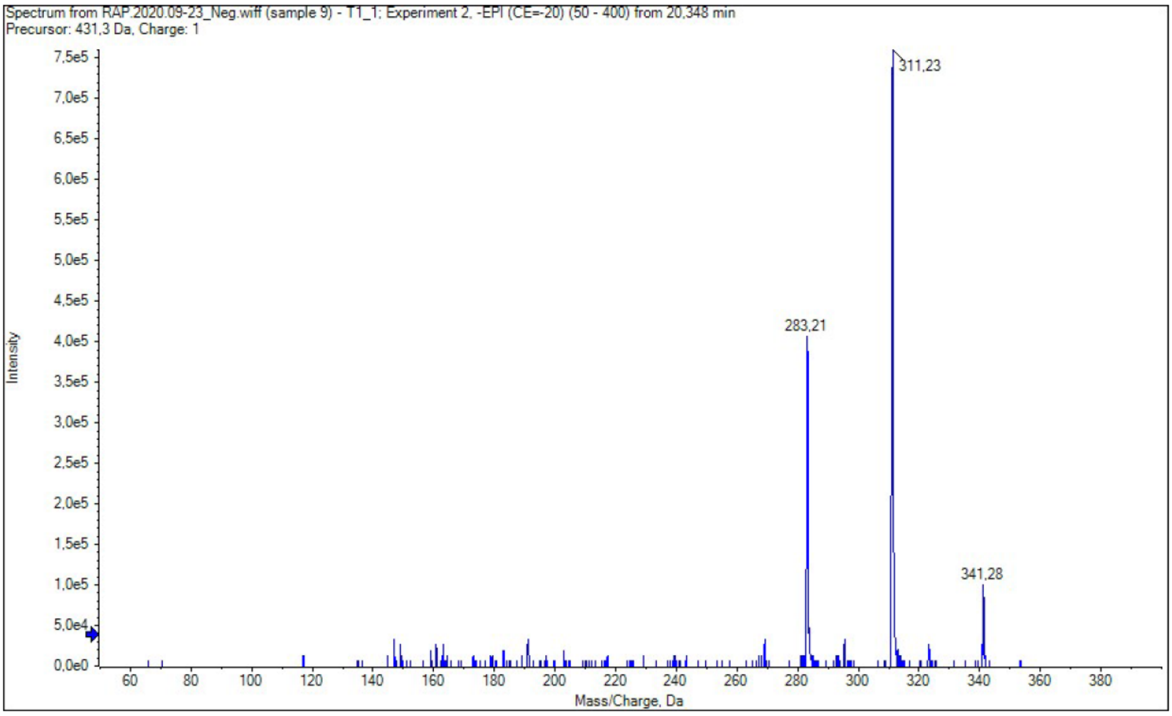

Figure S8. The fragmented mass spectrum obtained from flavonoid-rich *M. oleifera* leaf extract (MOL-Flav) in the negative ion mode of peak Nº **4** (*m/z* 431) by HPLC–QTRAP-MS/MS analysis related to the compound apigenin hexose isomer I (vitexin).

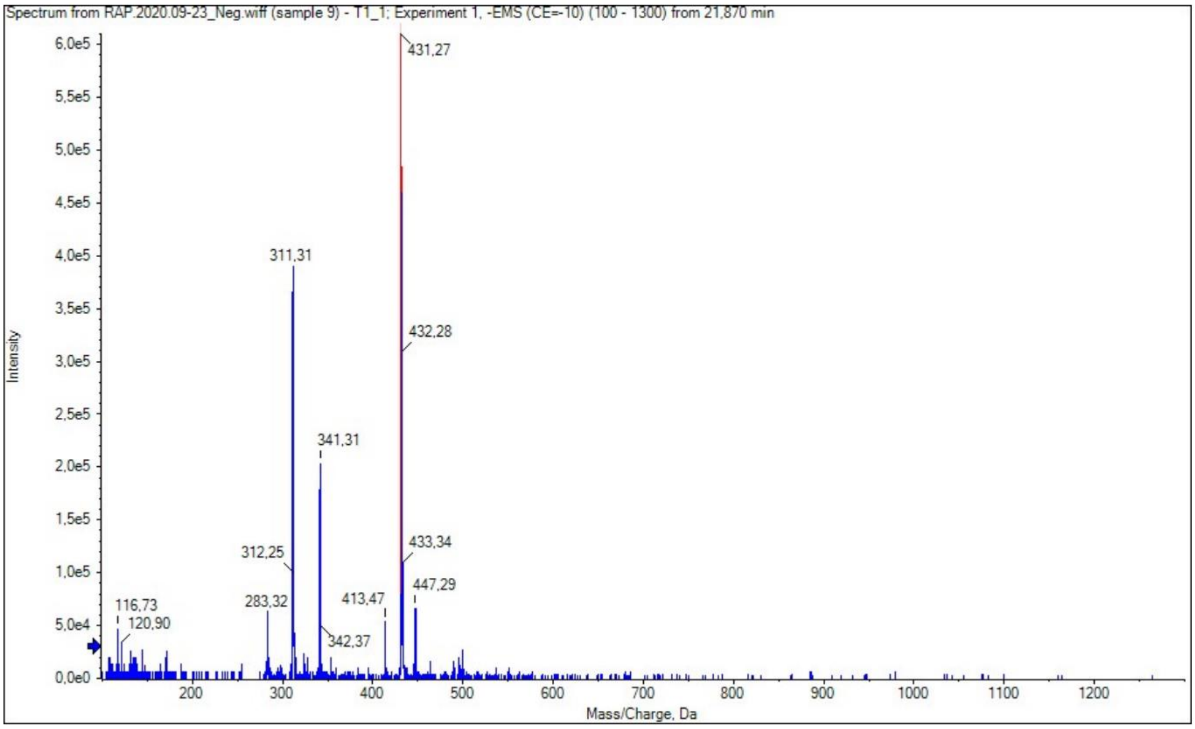

Figure S9. The mass spectrum obtained from flavonoid-rich *M. oleifera* leaf extract (MOL-Flav) in the negative ion mode of peak Nº **5** by HPLC–QTRAP-MS analysis related to the compound apigenin hexose isomer II (isovitexin).

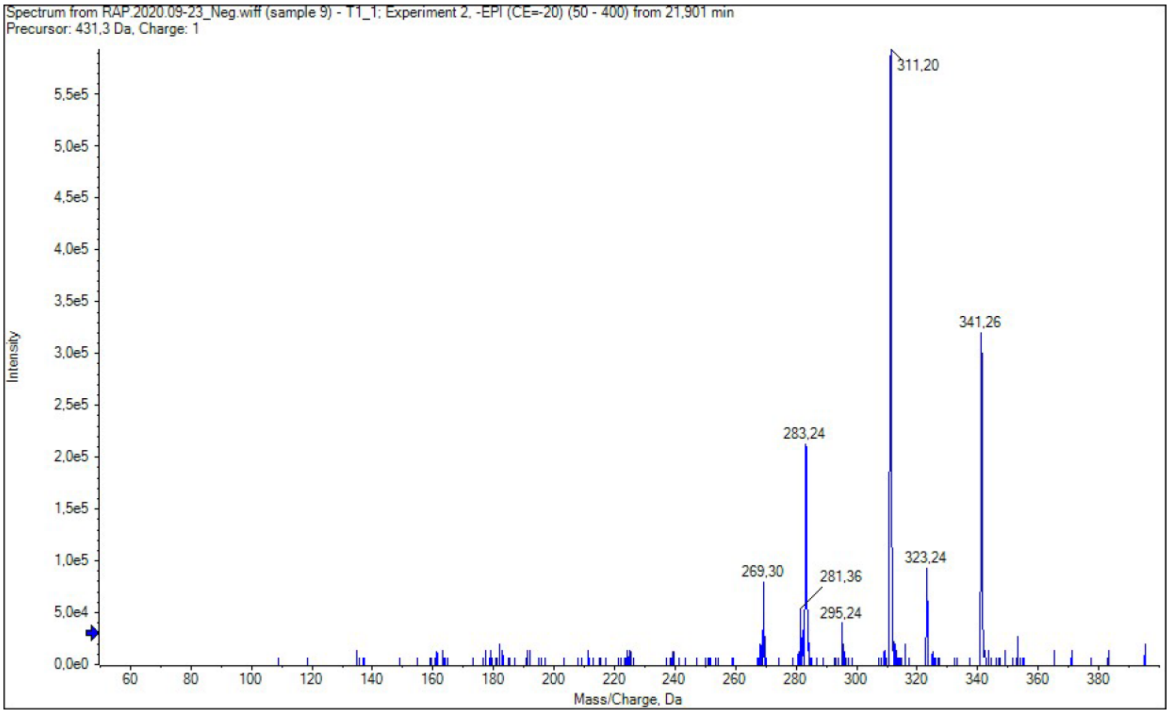

Figure S10. The fragmented mass spectrum obtained from flavonoid-rich *M. oleifera* leaf extract (MOL-Flav) in the negative ion mode of peak Nº **5** by HPLC–QTRAP-MS/MS analysis related to the compound apigenin hexose isomer II (isovitexin).

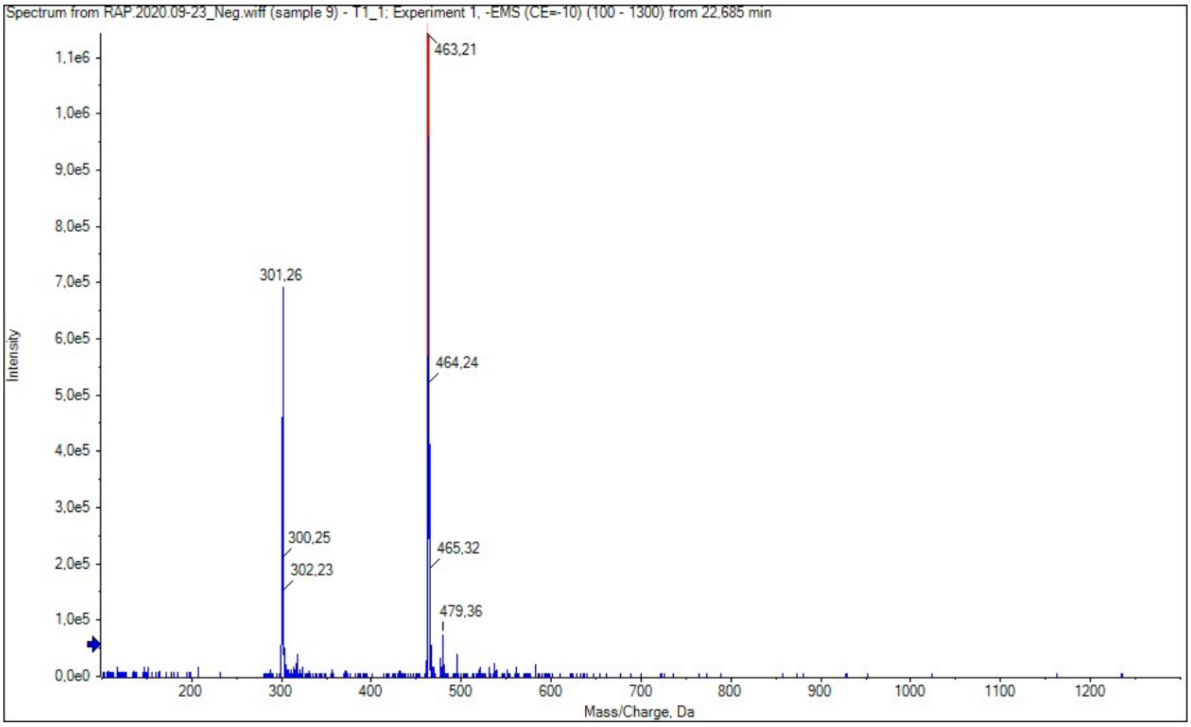

Figure S11. The mass spectrum obtained from flavonoid-rich *M. oleifera* leaf extract (MOL-Flav) in the negative ion mode of peak Nº **6** by HPLC–QTRAP-MS analysis related to the compound quercetin-3-*O*-hexose.

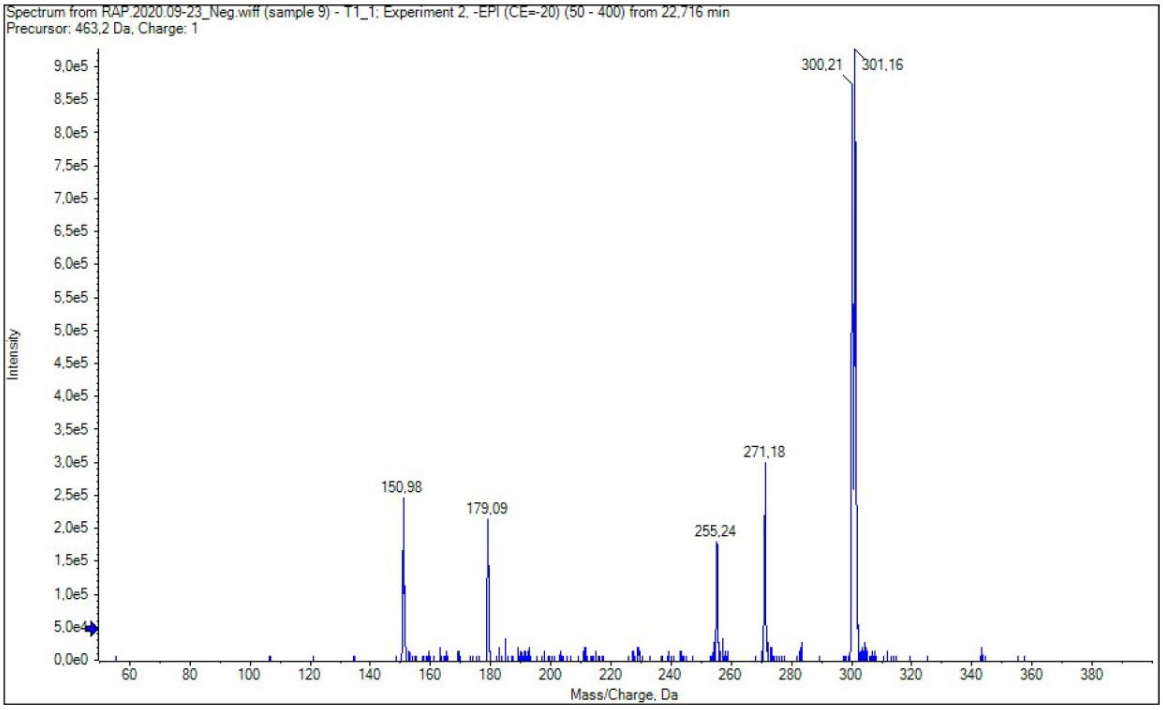

Figure S12. The fragmented mass spectrum obtained from flavonoid-rich *M. oleifera* leaf extract (MOL-Flav) in the negative ion mode of peak Nº **6** by HPLC–QTRAP-MS/MS analysis related to the compound quercetin-3-*O*-hexose.

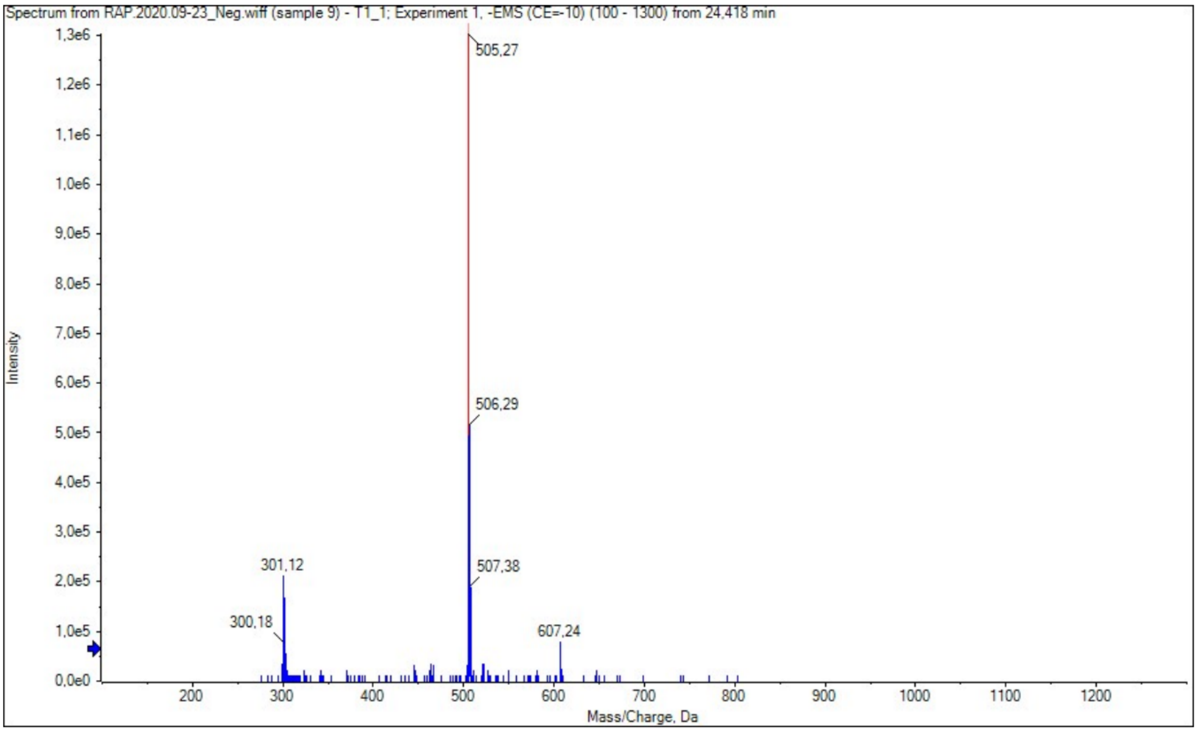

Figure S13. The mass spectrum obtained from flavonoid-rich *M. oleifera* leaf extract (MOL-Flav) in the negative ion mode of peak Nº **7** by HPLC–QTRAP-MS analysis related to the compound quercetin-3-*O*-acetyl-hexose.

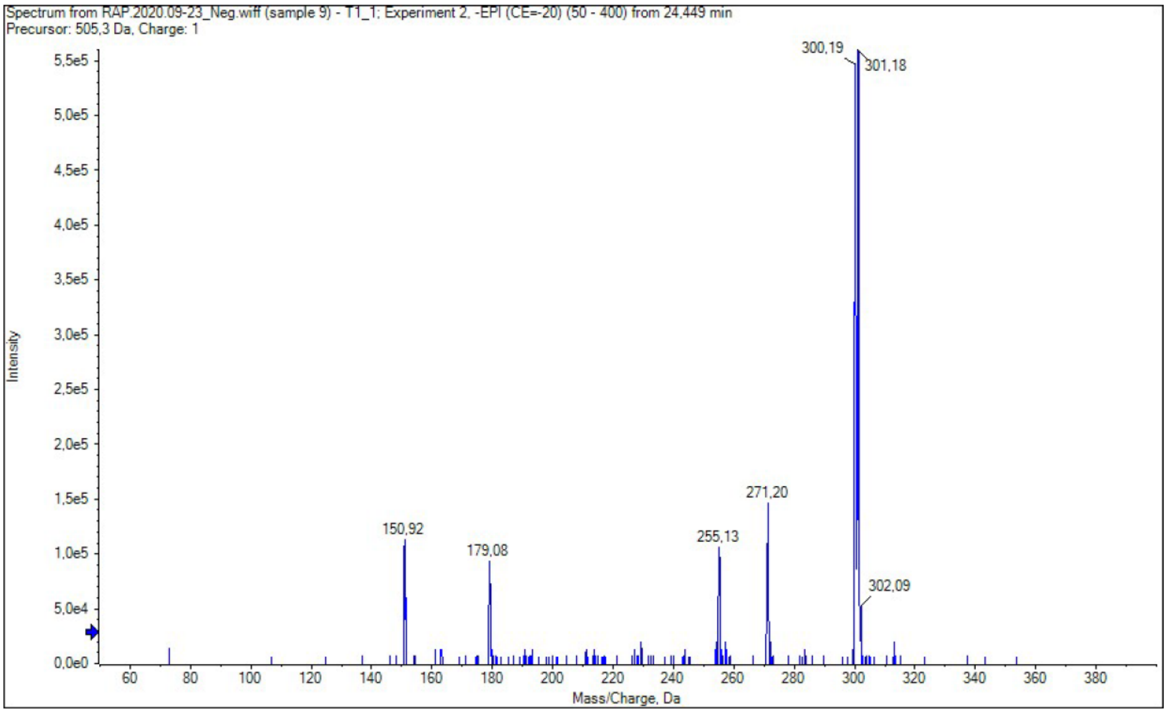

Figure S14. The fragmented mass spectrum obtained from flavonoid-rich *M. oleifera* leaf extract (MOL-Flav) in the negative ion mode of peak Nº **7** by HPLC–QTRAP-MS/MS analysis related to the compound quercetin-3-*O*-acetyl-hexose.

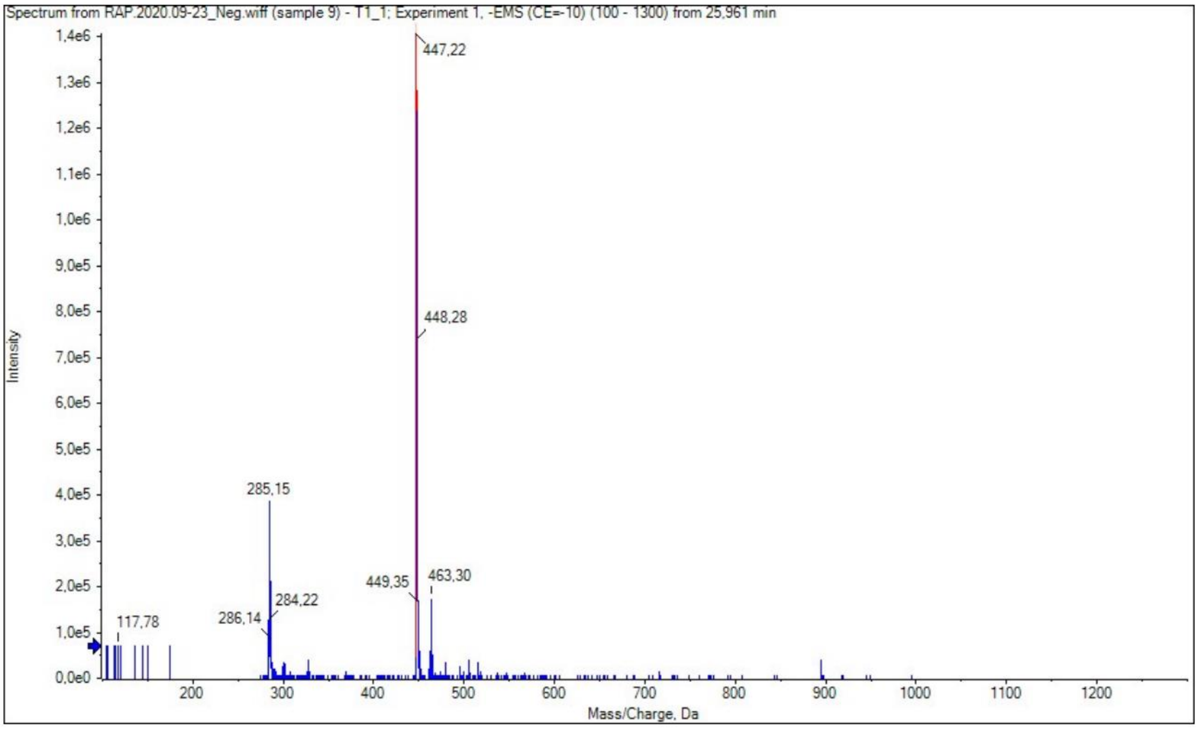

Figure S15. The mass spectrum obtained from flavonoid-rich *M. oleifera* leaf extract (MOL-Flav) in the negative ion mode of peak Nº **8** by HPLC–QTRAP-MS analysis related to the compound kaempferol-3-*O*-hexose.

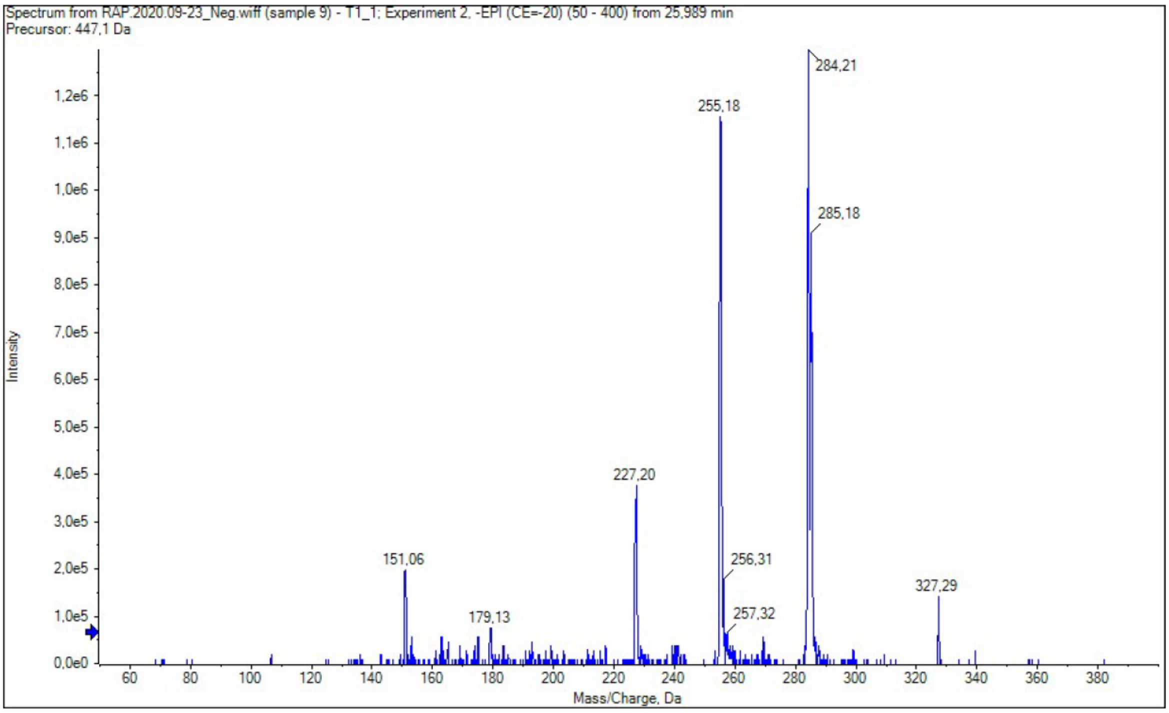

Figure S16. The fragmented mass spectrum obtained from flavonoid-rich *M. oleifera* leaf extract (MOL-Flav) in the negative ion mode of peak Nº **8** by HPLC–QTRAP-MS/MS analysis related to the compound kaempferol-3-*O*-hexose.

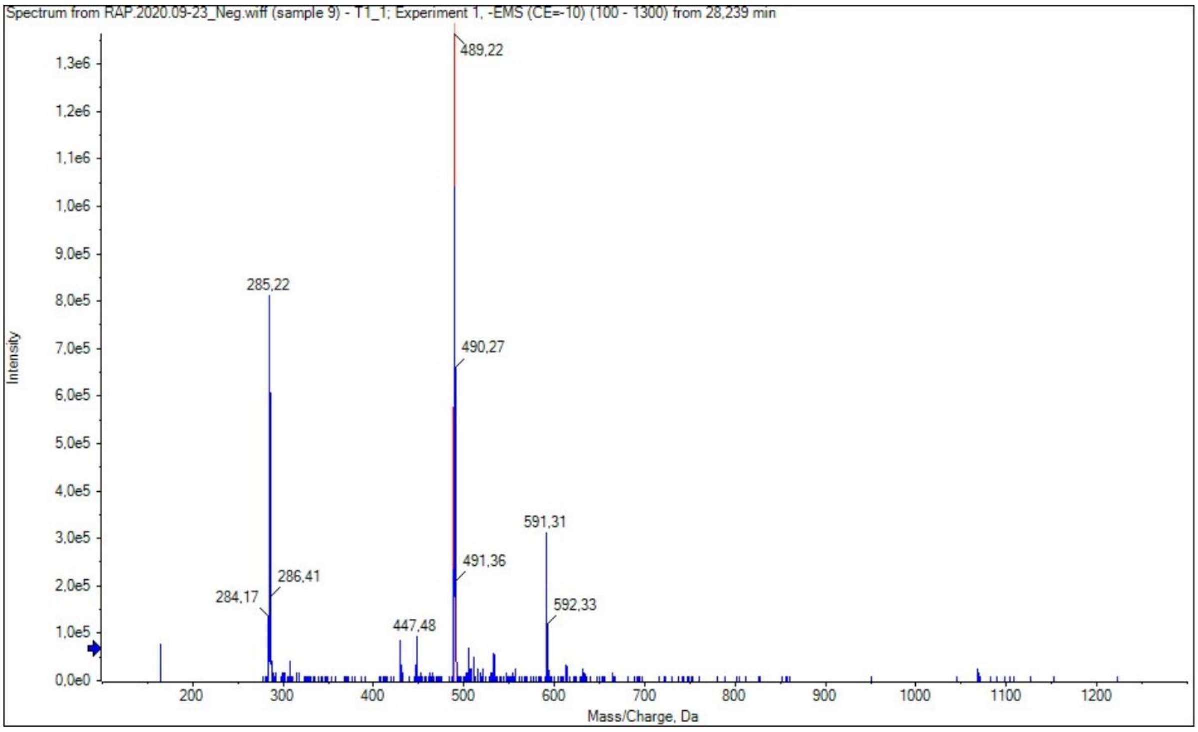

Figure S17. The mass spectrum obtained from flavonoid-rich *M. oleifera* leaf extract (MOL-Flav) in the negative ion mode of peak Nº **9** by HPLC–QTRAP-MS analysis related to the compound kaempferol-3-*O*-acetyl-hexose.

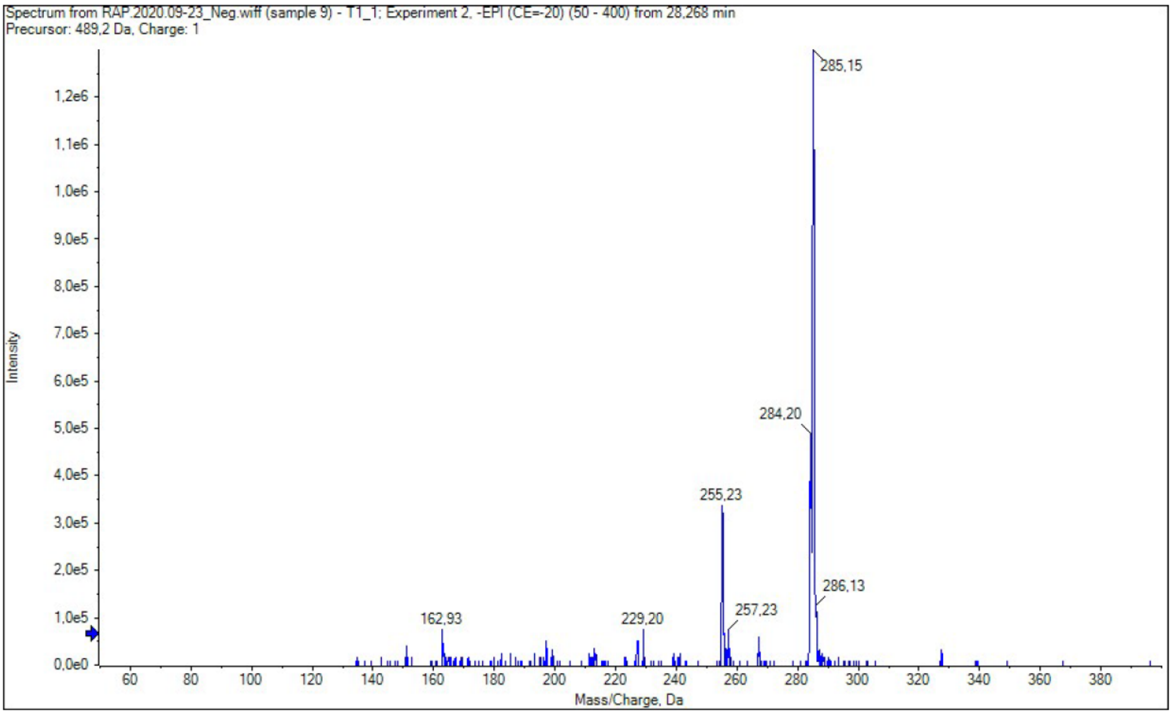

Figure S18 The fragmented mass spectrum obtained from flavonoid-rich *M. oleifera* leaf extract (MOL-Flav) in the negative ion mode of peak Nº **9** by HPLC–QTRAP-MS/MS analysis related to the compound kaempferol-3-*O*-acetyl-hexose.
